# Amyloid Deposits in a Bone Marrow Biopsy Alongside a Presumed Causative Clone

**DOI:** 10.1002/jha2.70180

**Published:** 2025-11-18

**Authors:** Rahul Banerjee, Marie K. Das, Kelly D. Smith

**Affiliations:** ^1^ Clinical Research Division Fred Hutchinson Cancer Center Seattle Washington USA; ^2^ Department of Medicine University of Washington Seattle Washington USA; ^3^ Department of Pathology & Laboratory Medicine University of Louisville School of Medicine Louisville Kentucky USA; ^4^ Department of Laboratory Medicine and Pathology University of Washington Seattle Washington USA

**Keywords:** AL amyloidosis, amyloidosis, ATTR amyloidosis, bone marrow

1

A 73‐year‐old male presented with worsening exertional dyspnea. Transthoracic echocardiography showed normal left ventricular size, preserved left ventricular ejection fraction (55%–60%), severe biatrial enlargement, and a dense speckling pattern. The left ventricular posterior wall measured 2.1 cm and the intraventricular septum measured 2.6 cm during diastole. Given that diastolic wall thicknesses over 1.2 cm can be seen in cardiac amyloidosis [1], this raised suspicion for an infiltrative process rather than more common processes such as hypertensive cardiomyopathy. Indeed, cardiac magnetic resonance imaging (top left) showed late gadolinium enhancement consistent with cardiac amyloidosis.

The patient denied any neuropathy and no pedal edema was noted on examination. While serum protein electrophoresis (SPEP) and serum immunofixation were unremarkable, serum kappa free light chain (FLC) was elevated at 54.4 milligrams per liter (mg/L, upper limit of normal [ULN] 19.4 mg/L). The serum lambda FLC was 8.5 mg/L (ULN 26.3 mg/L), yielding an abnormally elevated FLC ratio of 6.4 (reference range: 0.26–1.65). Troponin I was 0.041 nanograms per milliliter (ng/mL, ULN 0.110 ng/mL), but N‐terminus pro B‐type natriuretic peptide (nt‐proBNP) was significantly elevated at 4510 picograms per milliliter (pg/mL, ULN 125 pg/mL). Immunohistochemistry for kappa light chain on a trephine bone marrow (BM) core biopsy revealed that the majority of plasma cells and small lymphocytes were kappa‐restricted (top right). Congo red staining of the same specimen was positive with green birefringence in periosteal tissue (bottom left). Flow cytometry showed an abnormal kappa‐restricted population (bottom right) of CD5‐variable CD10‐negative B cells phenotypically consistent with marginal zone lymphoma (MZL). No *MYD88* or *CXCR4* mutations were noted.

Given the presence of Congo red positivity with a presumed causative clone within the same biopsy sample, a diagnosis of AL amyloidosis secondary to MZL was made. While rare, this entity has been reported [2]. The patient was immediately scheduled to begin rituximab and bendamustine, given his worsening symptoms; however, he opted to defer treatment to seek a second opinion at our center. We performed amyloid typing using liquid chromatography with tandem mass spectrometry (using methods described previously) [3], which demonstrated ATTR (transthyretin)‐type amyloidosis. The patient was instead started on the transthyretin stabilizer tafamidis and experienced gradual improvement in his dyspnea.

This case reinforces the importance of typing amyloidosis in virtually all cases. Late gadolinium enhancement and an FLC ratio over 5.0 are collectively quite suggestive of AL amyloidosis [4], especially when a lymphoproliferative clone is identified simultaneously. However, over 10% of cases of cardiac amyloidosis with abnormal SPEP/FLC results and BM amyloid deposits are actually due to ATTR deposition [5]. Periosteal congophilic deposits, although more common in ATTR amyloidosis, can be seen in AL amyloidosis as well [5]. While the desire to initiate treatment quickly is understandable for symptomatic patients, a complete diagnostic workup is mandatory given the high frequency of concurrent abnormal SPEP/SFLC results in older adults with ATTR amyloidosis (Figure 1). In our case, a brief delay for amyloid typing led to initiation of the correct treatment without unnecessary exposure to the potentially serious toxicities of alkylating agents such as bendamustine [6].

**FIGURE 1:**
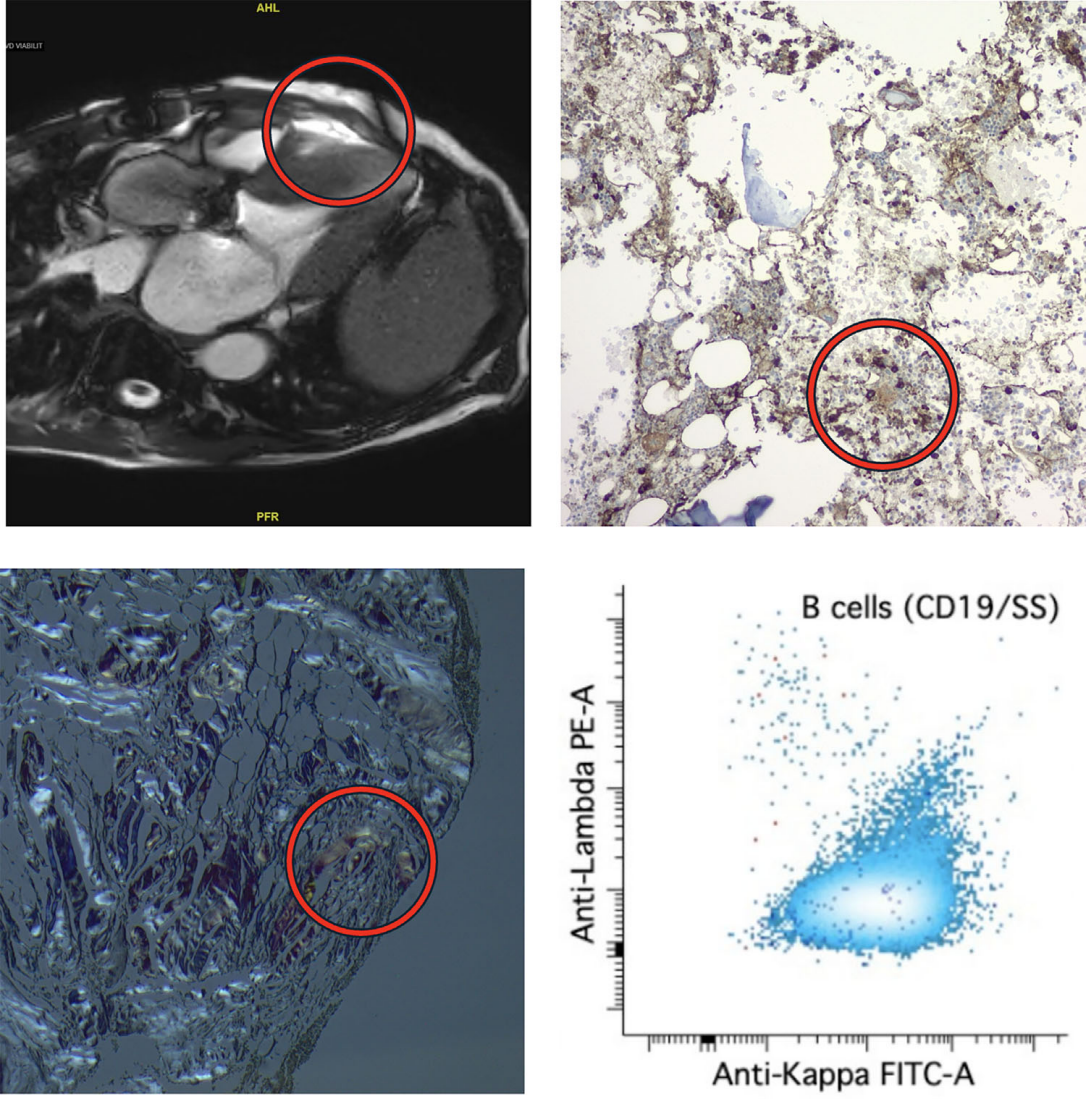
Workup for a patient with cardiac amyloidosis.

## Author Contributions

R.B. wrote the first draft. R.B., M.K.D., and K.D.S. provided critical feedback and approved the final manuscript.

## Funding

The authors have nothing to report.

## Consent

The patient provided consent for de‐identified discussion of his case.

## Conflicts of Interest

R.B. reports consulting: Abbvie, Adaptive Biotech, BMS, Caribou Biosciences, Genentech, Gilead/Kite, GSK, Janssen, Karyopharm, Legend Biotech, Pfizer, Poseida Therapeutics, Sanofi, SparkCures; Research: Abbvie, BMS, Janssen, Novartis, Pack Health, Prothena, Sanofi. The other authors declare no conflicts of interest.

## Data Availability

The authors have nothing to report.
